# Mapping water sources and access to drinking water in the Lake Chad region of Cameroon: a cross-sectional study

**DOI:** 10.11604/pamj.2023.46.98.42146

**Published:** 2023-12-08

**Authors:** Collins Buh Nkum, Michael Saah Fopa, Landry Beyala, Ketina Hirma Tchio-Nighie, Etienne Guenou, Aude Nanfak, Charlette Nangue, Jerome Ateudjieu

**Affiliations:** 1 Department of Health Research, *Meilleur Accès aux Soins de Santé (M.A. Santé)*, Yaoundé, Cameroon,; 2 Department of Public Health, Faculty of Medicine and Pharmaceutical Sciences, University of Dschang Cameroon, Dschang Cameroon,; 3 Division of Health Operations Research, Cameroon Ministry of Public Health, Yaoundé, Cameroon

**Keywords:** Water source, Lake Chad, Cameroon

## Abstract

**Introduction:**

people's access to quality water resources significantly improves their health. In Cameroon, access to drinking water is still limited and unequally distributed over the national territory with alarming figures in the northern part of the country. This study aimed to assess the distribution of water points and characterise water storage and treatment practices in households of the Lake Chad region of Cameroon.

**Methods:**

we conducted a cross-sectional descriptive study in Goulfey, Mada, and Makary health districts of the Far North Region of Cameroon from December 2013 to February 2014. Data were collected in face-to-face interviews with a structured questionnaire to assess household water behaviour and an observational grid for the characterisation of water points.

**Results:**

we identified a total of 303 water points, out of which 288 were assessed. Of these, 29.5% (85/288) were non-functional with functional failure observed as the main reason (47.6%). Of the 531 households reached, 527 (99.2%) were interviewed. Most households (70.2%) used boreholes as their main water source and only 3% of households used lakes as drinking water. The majority of households (90.4%) used clay pots for water storage within their homes. Buckets with covers are used in 21 (4.0%) while only 1 (0.2%) household used buckets without covers. Only 138 (26.2%) households treat their water and the main treatment method used is chlorination (89.1%).

**Conclusion:**

this study provides further evidence that access to safe water remains a real problem in the Lake Chad Basin. Therefore, interventions are needed to address the problem, but further studies are needed to strengthen its implementation.

## Introduction

Safe water is water that does not contain pathogens or chemical agents in concentrations that can harm health [1]. Accessibility of water in sufficient quantity and quality to meet basic human needs is a prerequisite for improved health and sustainable development [2]. The level of access to drinking water depends on a number of factors including low levels of education, climatic conditions, low household income, and urbanization, level of development, population growth, and the level of pollution [3]. Globally, several interventions are being made to improve the accessibility of drinking water. In this context, the United Nations has set a goal of providing access to safe and affordable water to all individuals worldwide by 2030 [4,5]. To reach this target, the Cameroon government; through the Strategy Document of Growth and Employment 2010-2020; intended to improve to 75% in 2020 the rate of access to safe drinking water with specific objectives: i) rehabilitate existing infrastructures carried out for the most part for more than 20 years; ii) carry out network extensions existing ones that have not kept pace with urban and demographic expansion; iii) promote the implementation of large-scale connection programs, and; iv) Cameroonian government, through the Ministry of Energy and Water, is developing national plans and strategies for water supply, and is setting up water exploration, research and exploitation in the cities and countryside [6,7].

Despite these various interventions, access to drinking water remains limited in Cameroon and is unevenly distributed [8-11]. A study reported that half of the population did not have access to an improved water source, and 80% of them lacked improved toilets [12]. The northern part of the country is threatened by extreme water scarcity and high climate variability. This situation and distribution of access to safe water is reflected in the distribution of water-related diseases including diarrheal diseases which are still a major cause of consultation in Cameroon particularly in the far north region [8]. Furthermore, Cameroon is frequently afflicted by cholera epidemics with high burdens of cases and deaths distributed mainly in the coastal zones of Cameroon (Littoral, South, and South West regions of Cameroon) and the Far North region of Cameroon.

In order to contribute to the reduction of mortality and morbidity due to diseases related to inadequate access to water, we proposed to in one hand to assess the distribution of water points, in the other hand, this study will characterise water storage and treatment practices in households of the Lake Chad region of Cameroon.

## Methods

**Study design:** this was a cross-sectional descriptive study, based on face-to-face administration of a pretested questionnaire in households and observation of water points using an observation grid. Data was collected from December 2013 to February 2014 by trained surveyors to assess water storage and treatment practices in households.

**Study setting:** this study was conducted in three health districts (Goulfey, Mada and Makary) around Lake Chad zone, which cover 6 sub divisions of the Logone and Chari division, in the Far North Region of Cameroon. These districts were among the most affected health districts during previous cholera outbreaks in Cameroon. The main economic activities are agriculture, fishing, cattle and sheep rearing. These health districts are subdivided into 26 health areas with health coverage provided by 29 health facilities [13].

**Study population:** this study targeted households of the health districts of Goulfey, Mada and Makary around Lake Chad and all water points in the communities of the targeted health districts. Water from unprotected water point were considered unimproved [14].

**Sampling procedure:** water points were exhaustively targeted in the 3 health districts.

**Sample size:** the minimum sample size determined was estimated at 397 households assuming a precision of 10%, a 95% confidence interval, a design effect of 2.0 [9], the average size (number of persons per household) of 5.4 in Cameroon according to the National Institute of Statistics, the prevalence of access to drinking water in the Far North Region was 28.3% in 2010 (r=0.283) [15] with an expected rate of non-response at 10% [16].

**Data collection procedures:** in each health district, a community health worker was selected to guide the data collection team to the selected communities. In each community, a permission was obtained from the community head before data collection in the community. We randomly determined with a bottle the direction to take from the centre of the village, the first household was randomly chosen, one household was systematically skipped and we recruited the next.

At the level of each household, the head of the household was informed about the objectives, the procedures, the benefits and the risks of the study before enrolment in the study. Those consenting to participate were administered a face-to-face interview questionnaire by trained surveyors. We identified, listed and categorized all points in each targeted health district with the help of community volunteers. The sanitary inspection of the water points allowed for the identification of potential sources of contamination at the various water points. This was done by observing the structure and its direct environment. The distance between the structure and certain sources of contamination was assessed using a tape measure. Additional information such as causes of non-functionality were requested from the village chief or a person able to answer.

**Data quality control:** data collection forms completed during the day were reviewed for inconsistencies, errors and missing variables and corrected at the end of each day. Data entry was carried out after the collection was completed. Data entry was done by the double entry method and cross referencing of databases to ensure data quality.

**Statistical analyses:** the software used for this activity was Epi Info from the Centers for Disease Control and Prevention (CDC) version 3.5.4. Key variables collected and we estimated the proportions of type of water points, functionality of water points, and quality of water points, type of water used by households, treatment and water storage.

**Funding:** this work was funded by *Meilleur Accès aux Soins de Santé (M.A. Santé)*.

**Ethics statement:** the ethical approval was obtained from the Cameroon National Ethics Committee for Human Health Research (No2013//384L/CNERSH/SP). Every participant was fully informed about the objectives, procedures, risks and benefits of the study, and only those who provided their verbal consent were included in the study.

## Results

**Characteristics of the surveyed water points:** a total of 303 water points were assessed among which 31 were in Goulfey, 137 in Mada, and 135 in Makary health districts. The most prevailing water source in both Goulfey and Makary was human-powered drillings (74.2% and 52.2% respectively) contrary to Mada which was predominated by hollow wells (38.7%). Table 1 presents the distribution of water point types per health district.

**Table 1:** distribution of water points surveyed at the Goulfey, Mada and Makary health districts of Cameroon

Water points	Goulfey, (N = 31)	Mada, (N = 137)	Makary, (N = 135)	Overall, (N = 303)
	n (%)	n (%)	n (%)	n (%[CI])
River Lake (RL)	1 (3.2)	11 (8.0)	3 (2.2)	15 (5.0 [2.9; 8.2])
Human powered drilling (HPD)	23 (74.2)	51 (37.2)	71 (52.6)	145(47.9 [42.1; 53.6])
Well with pump (WWP)	1 (3.2)	19 (13.9)	8 (5.9)	28 (9.2 [6.3; 13.2])
Hollow well (HW)	6 (19.4)	53 (38.7)	53 (39.3)	112 (37.0 [31.6; 42.7])
Drilling without pump (DWP)	0 (0.0)	3 (2.2)	0 (0.0)	3 (1.0 [0.3; 3.2])

**Functionality of the water sources:** among the 145 human-powered drilling (HPD) inspected, 41 (28.3%) were non-functional, similarly, of the 112 hollow well (HW) listed, 35 (31.2%) were also non-functional and 28 well with pump (WWP) surveyed, 9 (32.1%) were non-functional (Table 2).

**Table 2:** distribution of functionality of the water points at the Goulfey, Mada and Makary health districts of Cameroon

Water points	Functional, N = 203	Not functional, N = 85	Overall, N = 288
	n (%)	n (%)	n (%)
Human powered drilling (HPD)	104 (71.7)	41 (28.3)	145 (100.0)
Hollow well (HW)	77 (68.8)	35 (31.2)	112 (100.0)
Drilling without pump (DWP)	3 (100.0)	0 (0.0)	3 (100.0)
Well with pump (WWP)	19 (67.9)	9 (32.1)	28 (100.0)

**Reasons for non-functioning water points:** the reasons for the non-functionality of water points are presented in Table 3. Functional failure was observed as the main reason 40 (47.6%), followed by the presence of dried water source 19 (22.6%).

**Table 3:** reasons for non-functioning water points observed at the Goulfey, Mada and Makary health districts of Cameroon (N = 84)

Reasons for non-functioning water points	n (%)
Poor water quality	16 (19.0)
Collapse	4 (4.8)
Best water sources	3 (3.6)
Functional failure	40 (47.6)
Dried water source	19 (22.6)
Vandalism	2 (2.4)

**Quality of the water sources:** stagnant pools of water around the watering hole within 2 m were present in 80 (77.7%) HPD, 15 (78.9%) of WWP, 36 (46.8%) in HW and in all drilling without pump (DWP). A pond, garbage heap, sheep pen, or animal pen around the watering hole within 10 m was observed in all the water sources (N=200). Table 4 presents the functional water points and characteristics per point. Almost half 32 (41.6%) of the sampled HPD had an impermeable platform around that limits surface water infiltration. More than 94% of HW had an intact well seal and had a well-covered above floodwaters.

**Table 4:** quality of the water sources observed at the Goulfey, Mada and Makary health districts of Cameroon

		HPD		DWP		WWP		HW	
Observations of activities near water point	N	n (%)	95% CI	n (%)	95% CI	n (%)	95% CI	n (%)	95% CI
Latrine within 10 m	126	9 (8.7 3)	[4.3; 16.2]	2 (66.7)	[12.5; 98.2]	3 (15.8)	[4.2; 40.5]	NA	NA
Stagnant pools of water around the watering hole within 2 m	202	80 77.7)	[68.2; 85.0]	3 (100.0)	[31.0; 100.0]	15 (78.9)	[53.9; 93.0]	36 (46.8)	[35.4; 58.4]
Impermeable surface around the water point	202	99 (96.1)	[89.8; 98.7]	0 (0.0)	[0.0; 69.0]	1 (5.3)	[0.3; 28.1]	32 (41.6)	[30.6; 53.4]
Drainage system around the water point	200	84 (82.4)	[73.3; 88.9]	2 (66.7)	[12.5; 98.2]	5 (27.8)	[10.7; 53.6]	27 (35.1)	[24.8; 46.9]
Cracks in the subgrade that allow surface water to seep through	146	53 (53.0)	[42.8; 63.0]	0 (0.0)	[0.0; 94.5]	1 (33.3)	[1.8; 87.5]	18 (42.9)	[28.1; 58.9]
Pond, garbage heap, sheep pen, or animal pen around the watering hole within 10 m	200	54 (52.9)	[42.8; 62.8]	3 (100.0)	[31.0; 100.0]	11 (61.1)	[36.1; 81.7]	33 (42.9)	[31.8; 54.6]
Barrier restricting pet access to the watering hole	201	14 (13.6)	[7.9; 22.1]	0 (0.0)	[0.0; 69.0]	2 (11.1)	[1.9; 36.1]	7 (9.1)	[4.0; 18.4]
Well covered	76	72 (94.7)	[86.4; 98.3]	NA	NA	NA	NA	NA	NA

HPW: ; DWP: drilling without pump; WWP: well with pump; HW: hollow well; NA: not applicable

**Water access in households:** of the 535 households planned, 531 (99.2%) were visited and 527 were interviewed, for a non-response rate of 0.7%. The households interviewed were distributed as follows: 104 households in the Goulfey HD (19.8%); 176 households in the Mada HD (33.4%) and 246 households in the Makary HD (46.8%). About 75% of these households drink water from unimproved sources. The most common water source used was community boreholes, representing 70.2%. Table 5 gives the distribution of water points used in households. Water was stored in all the households, 490 (90.4%) used clay pots for water storage at home. Bucket with covers is used in 21 (4.0%) while only 1 (0.2%) household used buckets without covers.

**Table 5:** distribution of water usage and storage practices in households of the Goulfey, Mada and Makary health districts of Cameroon (N=527)

	Goulfey, N = 104	Mada, N = 176	Makary, N = 247	Overall, N = 527
	n (%)	n (%)	n (%)	n (%[CI])
**Water storage practices in households**				
Bottles	0 (0.0)	4 (2.3)	0 (0.0)	4 (0.8 [0.3; 2.0])
Buckets with cover	0 (0.0)	15 (8.5)	6 (2.4)	21 (4.0 [2.6; 6.0])
Buckets without cover	0 (0.0)	1 (0.6)	0 (0.0)	1 (0.2 [0.0; 1.3])
Can	0 (0.0)	8 (4.5)	3 (1.2)	11 (2.1 [1.2; 3.7])
Cistern	0 (0.0)	3 (1.7)	4 (1.6)	7 (1.3 [0.6; 2.7])
Clay pot	104 (100.0)	151 (85.8)	235 (95.1)	490 (93.0 [90.4; 94.9])
**Water used in households**				
CDE (Camerounaise des Eaux)	0 (0.0)	1 (0.6)	24 (9.7)	25 (4.7 [3.1; 6.8])
Wells	0 (0.0)	21 (11.9)	7 (2.8)	28 (5.3 [3.4; 7.2])
Boreholes	97 (93.3)	122 (69.3)	151 (61.1)	370 (70.2 [66.8; 74.6])
River	46 (44.2)	28 (15.9)	10 (4.0)	84 (15.9 [13.2; 19.5])
Lake	0 (0.0)	17 (9.7)	1 (0.4)	18 (3.4 [2.2; 5.4])

**Water treatment methods:** Table 6 presents the water treatment methods used in households. Among the 527 households surveyed, only 138 (26.2%) households treated their water. The main treatment methods used are chlorination (89.1%), decantation (6.5%) and filtration (3.6%).

**Table 6:** distribution of water treatment methods used in households of the Goulfey, Mada and Makary health districts of Cameroon (N = 138)

Water treatment methods	Goulfey, N = 29	Mada, N = 67	Makary, N = 42	Overall, N = 138
	n (%)	n (%)	n (%)	n (%[CI])
Decanting	0 (0.0)	7 (10.4)	2 (4.8)	9 (6.5 [3.40; 12.1])
Filtration	0 (0.0)	2 (3.0)	3 (7.1)	5 (3.6 [1.50; 8.50])
Chlorination	28 (96.6)	60 (89.6)	35 (83.3)	123 (89.1 [82.7; 93.4])
Boiling	1 (3.4)	0 (0.0)	3 (7.1)	4 (2.9 [1.1; 7.6])
Solar disinfection	0 (0.0)	0 (0.0)	0 (0.0)	0 (0.0 [NA])

## Discussion

A total of 303 water points were assessed in the Goulfey, Makary and Mada health districts (Table 1). The most prevailing water point in both Goulfey HD and Makary HD were human powered drillings (74.2% and 52.2% respectively) contrarily to Mada HD which was predominated by hollow well (38.7%). Drilling without pump was the less common water point and was found in Mada HD (2.2%).

Our results show that human powered drilling (boreholes) was the most observed water point (47.9%). This result is different from those of Ndjama *et al*. who reported 16% boreholes in the Ngoua and Bobongo basins of Douala City in March 2007 as the most common water point [17]. The difference observed here could be explained by the fact that this study was carried out an urban area with a different climatic context from that of the Lake Chad basin area. The use of boreholes in about 1/2 of the households shows that this water supply facility is socially and culturally accepted by the population.

About one third of the boreholes surveyed (28.3%) were non-functional and a pump failure was the cause of the malfunction in 47.6% of cases. Most of the studies focused on water supply do not describe the functionality of water points and particularly boreholes. This result raises the problem of maintenance of water extraction works. The work of Kumpel *et al*. [18], in sub-Saharan Africa comes to the same conclusion. Indeed, he notes that community-based water supply structures (boreholes, wells, etc.) generally have inadequate maintenance. This problem of management of community water supply points evokes irregularities in the implementation of the directives of the national policy on drinking water supply and rural sanitation of the Ministry of Energy and Water, which states that in order to ensure the sustainability of drinking water supply infrastructures, the management, maintenance and upkeep of water extraction equipment are the responsibility of the beneficiaries [19].

The problem of water point management also influences the quality of functional sources. Indeed, among the improved and functional water source identified, 77.7% of the HPD had stagnant pools of water around the watering hole within 2 m, there was the presence of pond, garbage heap, sheep pen, or animal pen around the watering hole within 10m were among the hazard identified in 52.2% of HPD. More than 90% boreholes were well covered and capped (N=76), in accordance with a study reported in Port Harcourt, Nigeria by Kumpel *et al*. with more than 83% well-sealed boreholes and almost all (96.1%) of the surveyed boreholes had an impermeable surface around the water point this data was lower (47%) with that reported by Kumpel *et al*. [18].

The maintenance problem remains crucial and is at the origin of a wrong investment strategy based on a false distribution of drinking water supply works, the major consequence of which will be the proliferation of waterborne diseases within the affected communities. A focus on the rehabilitation and maintenance of existing water points would be more beneficial to the population and would ensure the sustainability of the water supply. The establishment of a trained and effectively functioning management committee in each community, working in collaboration with the decentralised territorial authorities will be necessary for the successful implementation of this directive.

**Access to drinking water**: improved drinking water sources are not accessible to all rural areas in Cameroon. In this study, many households (24.6%) are reported to be drinking water from unimproved and unsafe sources such as wells, rivers, and lakes, which can be due to the poor distribution of improved water sources across the country as a result of a direct consequence of the non-achievement of Millennium Development Goal (MDG) 7 to which Cameroon has subscribed through numerous policies [4]. However, only 4.7% of households had access to piped water from the national water distribution network, and most of those who did have access to safe water were supplied by boreholes (70.2%). We can even assume that this proportion may be lower, as the improvement of the water source does not mean that the water is safe, as it does not take into account the measured microbial quality of the water.

**Water storage at households**: water storage is a common and cultural practice in Cameroonian households as in many other countries [20,21]. Our study shows that water is stored in all households surveyed, irrespective of the source. People store water to meet supply challenges such as long distance to the water source and water scarcity. Stored water is used for drinking and other domestic needs. The containers used for storage vary from household to household as do the volumes. This is a key determinant in understanding the fate of water after collection and therefore in assessing its quality, especially when storage conditions are also assessed, as the risk of contamination can arise from storage conditions. Over 90% of the households surveyed used clay pots to store their water and less than 5% used buckets. The use of clay pots for water storage can expose the water to contamination by micro-organisms from the environment. The condition and state of their containers can also be sources of contamination for the stored water. There is a need to support and develop strategies to educate people, particularly at the community level, on good water collection, transport, storage and treatment practices. Though efforts are being made to improve water access and standardized water treatment methods could help to overcome some of the problems of quantity and quality. All of this can have a significant influence on reducing the prevalence of water-related diseases at the community level in the most vulnerable areas [20,21].

**Water treatment methods**: it was revealed from this survey that only 138 (26.2%) households believe that their water source is potable and among them, 123 (89.1%) use chlorine for water treatment at household. It helps us understand how far people know about water treatment methods and how many of them finally practice them.

**Limitations**: difficulties such as insecurity and difficult access in some localities did not allow for equitable coverage of all health districts. However, we worked in 17 of the 26 Health areas in the study zone, and the fact that not all HDs were equitably represented did not affect the validity of the data. Similarly, the use of translators in the data collection procedure could have led to an information bias due to the loss of the real meaning of the information. To address this, we chose a qualified interviewer who was fluent in the most commonly spoken language. During the training of the interviewers, we set up a procedure of double translation of the questionnaire by different people. Thus, the effect of translation cannot significantly influence the quality of the information produced by this study.

## Conclusion

The source of drinking water used is a good indicator of the quality of the water consumed by the household. It is therefore imperative that this source is developed. This is because improve water sources are less distributed compared to households needs and appropriate households water treatment and safe storage practices are less implemented and not sustainable enough to ensure population safe access to water. This study provides further evidence that access to safe drinking water remains a real problem that increases the prevalence of diarrhoeal diseases in the Lake Chad Basin. Therefore, a comprehensive and partnership-based programme is needed to tackle the problem, but further studies will be needed to strengthen its implementation. Adequate multisectorial intervention should be taken in other to ensure sufficient and safe water to population and those in remote areas. There is also a need for local authorities to constantly make adjustments of water coverage estimates to ensure normal supply and shortage-free water distribution network.

### What is known about this topic

- *Safe water is water that does not contain pathogens or chemical agents in concentrations that can harm health*;
- *The level of access to drinking water depends on a number of factors including low levels of education, climatic conditions, low household income, urbanization, level of development, population growth, and the level of pollution*;
- *Globally, several interventions are being made to improve the accessibility of drinking water*.

### What this study adds

- *This study provides further evidence that access to safe drinking water remains a real problem that increases the prevalence of diarrhoeal diseases in the Lake Chad Basin*;
- *Our study reveals that approximately one third of water points assessed in the Lake Chad Basin were non-functional, mainly due to functional failure*;
- *This study provides the intervention needs to improve water storage and treatment methods used in the Lake Chad Basin*.
